# Gold-Based Nanoplataform for the Treatment of Anaplastic Thyroid Carcinoma: A Step Forward

**DOI:** 10.3390/cancers13061242

**Published:** 2021-03-12

**Authors:** Mariana Amaral, Adília J. Charmier, Ricardo A. Afonso, José Catarino, Pedro Faísca, Lina Carvalho, Lia Ascensão, João M. P. Coelho, M. Manuela Gaspar, Catarina Pinto Reis

**Affiliations:** 1 Research Institute for Medicines (iMed.ULisboa), Faculty of Pharmacy, Universidade de Lisboa, 1649-003 Lisbon, Portugal; marianaamaral@campus.ul.pt (M.A.); mgaspar@ff.ulisboa.pt (M.M.G.); 2 DREAMS, Universidade Lusófona de Humanidades e Tecnologias, Campo Grande 376, 1749-024 Lisbon, Portugal; adilia.januario@ulusofona.pt; 3 Ciências Funcionais e Alvos Terapêuticos, NOVA Medical School Faculdade de Ciências Médicas (NMS|FCM), Universidade Nova de Lisboa, 1169-056 Lisbon, Portugal; ricardo.afonso@nms.unl.pt; 4 Departamento de Física, Faculdade de Ciências e Tecnologia, Universidade Nova de Lisboa, 2829-516 Caparica, Portugal; 5 Laboratório Veterinário, Faculdade de Medicina Veterinária—Universidade Lusófona de Humanidades e Tecnologias/DNAtech, 1749-024 Lisbon, Portugal; p5663@ulusofona.pt (J.C.); pedrofaisca@ulusofona.pt (P.F.); 6 Central Testing Laboratory, Campus de Santiago, University of Aveiro, 3810-193 Aveiro, Portugal; linamcarvalho@ua.pt; 7 Centro de Estudos do Ambiente e do Mar (CESAM), Faculdade de Ciências, Universidade de Lisboa, Campo Grande, 1749-016 Lisbon, Portugal; lmpsousa@fc.ul.pt; 8 Instituto de Biofísica e Engenharia Biomédica (IBEB), Faculdade de Ciências, Universidade de Lisboa, Campo Grande, 1749-016 Lisbon, Portugal; jmcoelho@fc.ul.pt

**Keywords:** anaplastic thyroid carcinoma, photothermal therapy, novel therapies, gold nanoparticles

## Abstract

**Simple Summary:**

Anaplastic thyroid carcinoma (ATC) is a subtype of thyroid cancer that generally does not respond to the available treatments, making it one of the most lethal cancers known. As an alternative, Photothermal Therapy (PTT) can be considered a promising strategy. In this case, gold nanoparticles (AuNPs) are activated when irradiated by light, enhancing the local temperature, resulting in cell death. Thus, this study aims to formulate AuNPs specifically for targeting PTT to ATC. For this, biocompatible AuNPs were designed and functionalized with ATC-specific ligands (holo-transferrin, epidermal growth factor (EGF), and lapatinib), showing optimal physicochemical properties. Safety, efficacy, and ATC specificity were determined in vitro. Holo-transferrin functionalized-AuNPs were the most effective for PTT against ATC. Safety and biodistribution were studied in vivo, proving that the formulation was safe for intratumoural administration. So, taking these results all together, AuNPs-mediated-PTT seems to be a promising hint to find a viable treatment for ATC.

**Abstract:**

Anaplastic thyroid carcinoma (ATC) is a very rare subtype of thyroid carcinoma and one of the most lethal malignancies. Poor prognosis is mainly associated with its undifferentiated nature, inoperability, and failing to respond to the typically used therapies for thyroid cancer. Photothermal Therapy (PTT) entails using light to increase tissues’ temperature, leading to hyperthermia-mediated cell death. Tumours are more susceptible to heat as they are unable to dissipate it. By using functionalized gold nanoparticles (AuNPs) that transform light energy into heat, it is possible to target the heat to the tumour. This study aims to formulate ATC-targeted AuNPs able to convert near-infrared light into heat, for PTT of ATC. Different AuNPs were synthetized and coated. Size, morphology, and surface plasmon resonances band were determined. The optimized coated-AuNPs were then functionalized with ligands to assess ATC’s specificity. Safety, efficacy, and selectivity were assessed in vitro. The formulations were deemed safe when not irradiated (>70% cell viability) and selective for ATC. However, when irradiated, holo-transferrin-AuNPs were the most cytotoxic (22% of cell viability). The biodistribution and safety of this formulation was assessed in vivo. Overall, this novel formulation appears to be a highly promising approach to evaluate in a very near future.

## 1. Introduction

Although thyroid cancer accounts for 2% of all cancer diagnoses, the very rare anaplastic thyroid carcinoma (ATC), an undifferentiated subtype of thyroid cancer, is one of the most lethal known malignancies, with a 90% mortality rate [1,2]. In fact, the poor survival prognosis of 3–5 months after diagnosis is associated not only with the extreme aggressiveness of this tumour, but also to its undifferentiated nature [1]. A reflection of the accentuated aggressiveness is its stage IV classification at diagnosis, regardless of tumour size, lymphatic nodes invasion, and metastatic status [3]. Thyroid carcinomas are generally treated with a combination of surgery, radioactive iodine, and thyroid hormone suppression therapy, but as ATC has lost thyroid-like features (i.e., iodine uptake and thyroid hormone production), it generally fails to respond to the previous mentioned treatments [4,5,6]. Although ATC is currently treated with a combination of debulking surgery, radio, and chemotherapy, treatment is carried out with palliative intents, as ATC has extremely low cure rates [7]. The mentioned multimodal treatment course slightly increases patients’ survival, up to 10 months, and thus there is the need to find effective therapies and improve survival rates as well as quality of life of these patients [3]. Currently, the research in ATC therapeutics has shifted to targeted and multitargeted therapies (i.e., small-molecule tyrosine kinase inhibitors), but, due to the adverse events associated to these treatment options, less invasive and alternative strategies should be explored [1,3,5,8].

Photothermal therapy (PTT) uses light/heat sources to increase the local temperature of tissues [9]. In comparison to the current cancer therapies, PTT presents many advantages, such as being minimally invasive with low toxicity and spatial-temporal specificity [10,11]. While healthy tissues can dissipate the heat generated from the light source, tumours have different vasculature, and consequently are unable to dissipate the generated heat, causing the cells to undergo irreversible damage. This is usually characterized by protein denaturation and disruption of the cellular membranes, culminating in hyperthermia-mediated cell death [10,12,13]. The main limitation of PTT is the weak penetration capacity of the radiation used, allowing only the treatment of very superficial and localized tumours [14]. Nanotechnology can help PTT to overcome these challenges through the use of photoabsorbent nanoparticles (NPs), changing the photothermal properties of the medium and enhancing tumour hyperthermia [15]. In order to better enhance PTT, NPs are required to present the following set of characteristics: absorption at the wavelengths of biological windows (650–1400 nm) (as at these wavelength intervals tissues’ light scattering and absorption is reduced), and conversion of light into thermal energy.

Gold NPs (AuNPs) are a type of inert inorganic NPs with interesting applications in nanomedicine due to their unique and versatile optical and physicochemical properties, biocompatibility, stability, and low cytotoxicity [16,17,18]. AuNPs have many advantages, including efficient light-heat conversion and tunable optical properties, that can be manipulated by varying the AuNPs physical characteristics such as size and shape [19,20,21]. The majority of the commercial AuNPs present surface plasmon resonances (SPR) bands at wavelengths below the biological window, in the UV or visible spectrum, which have very poor penetrative capacity into human tissues [22,23,24]. It is our aim that these AuNPs absorb in the near infrared (NIR) range of the light spectrum (650–950 nm), allowing the treatment of more deep solid tumours, localized up to 2–3 cm below the skin [25,26].

Since ATC is a relatively superficial tumour, it is a more obvious candidate for AuNPs-mediated NIR-PTT. Our group has previously developed a novel formulation of functionalized AuNPs, produced using a plant extract of *Plectranthus saccatus*. These AuNPs were then covered with a polymeric coating composed of Hyaluronic Acid and Oleic Acid (HAOA) to specifically treat melanoma [27,28,29]. To treat ATC, this formulation required several modifications by optimizing the previously formulated coated-AuNPs and by changing AuNPs’ surface with ligands. In terms of ATC cellular markers, both epidermal growth factor (EGF) receptor (EGFR) and type 1 transferrin receptor (TfR1/CD71) are membrane receptors overexpressed in this type of cancer. Thus, to maximize the NPs specificity, AuNPs were functionalized with EGF or lapatinib (to target EGFR), or holo-transferrin (HTf, to target TfR1/CD71). These novel AuNPs were characterized regarding its physicochemical properties (i.e., mean size and size distribution, conjugation efficiency, absorbance spectra, and morphology). The most appropriate ligand was then chosen regarding the results of different studies, such as the overall physicochemical properties of the functionalized and coated AuNPs and in vitro safety, selectivity, and efficacy. After optimization, the safety and biodistribution of the AuNPs were preliminary assessed in vivo, using CD-1 mice. This work aimed to produce small, monodisperse, and highly selective AuNPs to be targeted for ATC cells. These NPs must be biocompatible, not harm healthy tissues, and effective, with high cytotoxicity, when activated by laser, against ATC cells.

## 2. Materials and Methods

### 2.1. Materials

#### 2.1.1. Reagents

Gold (III) chloride trihydrate, Silver Nitrate, L-ascorbic Acid, Hyaluronic Acid, Rosmarinic Acid (RA), Oleic Acid, Holo-Transferrin human (powder, grade 97%), and Lapatinib (98%) were acquired from Sigma-Aldrich (St. Louis, MO, USA). EGF Recombinant Human Protein was supplied from Gibco (ThermoFisher Scientific, Waltham, MA, USA). Bradford Dye Reagent was purchased from Alfa Aesar (ThermoFisher Scientific, Waltham, MA, USA). Water MilliQ by Merck Millipore (Burlington, MA, USA). All chemical products and solvents used were of analytical purity grade.

#### 2.1.2. Cell Lines and Cell Culture

Cell growth was done according to supplier’s instructions (ATCC). The human keratinocyte cell line HaCat was maintained in Dulbecco’s modified Eagle’s medium (DMEM), with high-glucose (4500 mg/mL), complemented with 100 µg/mL penicillin/streptomycin, and 10% foetal bovine serum (FBS). Human ATC cell line 8505C was maintained in RPMI 1640 medium and supplemented as previously mentioned for DMEM. All cell lines were stored in a humidified chamber at 37 °C, in a 5% CO_2_ atmosphere. The cell cultures were maintained every 2 to 3 days, at which the medium was changed, until a confluence of 80% was achieved.

#### 2.1.3. Animals

Male CD1 mice were purchased from IHMT (Lisboa, Portugal). The animal housing was kept at a controlled temperature of 22.0 ± 4.0 °C, humidity at 50.0 ± 15.0%, and a cycle of light/dark of 12 h in Faculty of Pharmacy facilities. Animals were kept under standard hygiene conditions, fed commercial chow, and given acidified drinking water ad libitum.

All experiments regarding the use of animals were conducted in accordance with the EU Directives (2010/63/UE), the Portuguese Law (DL 113/2013, 2880/2015 and 260/2016), and were approved both by the Animal Welfare Body (ORBEA) of the Faculty of Pharmacy, University of Lisbon, and of the Ethics Committee of the NOVA Medical School Faculdade de Ciências Médicas, Universidade NOVA de Lisboa.

### 2.2. Methods

#### 2.2.1. Preparation of HAOA-Coated AuNPs

The HAOA-coated AuNPs were prepared using a modified approach previously published by our group [27]. Briefly, an aqueous solution of gold (III) chloride trihydride was mixed with an aqueous solution containing silver nitrate, L-ascorbic acid, and RA. The mixture was continuously stirred for 15 min (800 rpm, Heidolph magnetic stirring hotplate MR 3001, Heidolph Instruments, Schwabach, Germany) at room temperature. Two different ratios of gold:RA (m/m) were tested, 5:3 and 5:10, originating two different AuNPs cores.

The HAOA coating was prepared by adding to Milli-Q water, HA, OA, and sodium hydroxide, at 60 °C and under a continuous stirring at 400 rpm. This aqueous mixture is stirred overnight at the mentioned conditions. In order to coat the AuNPs’ core, the HAOA prepared overnight is cooled to room temperature, and was mixed with the AuNPs’ core solution, 1:1 (*v*/*v*). The method of functionalization of the HAOA-coated AuNPs was the same regardless of the ligand (i.e., EGF, HTf, lapatinib) by mixing the HAOA-coated AuNPs with the phosphate buffered saline (PBS, USP32) solution containing the intended ligand under magnetic continuous stirring for 30 min at room temperature at a ratio of 1:1 (*v*/*v*).

To recover AuNPs, the suspension was centrifuged at 7200× *g* for 15 min (Beckman Instruments Centrifuge, Inc., Brea, CA, USA). The supernatant was stored to determine the efficiency of ligand conjugation to the NPs, and the correspondent pellet of the functionalized HAOA-coated AuNPs was suspended in PBS and stored at −4 °C.

#### 2.2.2. HAOA-Coated AuNPs Characterization

- Mean Size and Polydispersivity Index (PdI)

The different formulations, AuNPs core and HAOA-coated AuNPs, functionalized with the different ligands and non-functionalized, were characterized regarding its mean size and polydispersivity index (PdI). The formulations were diluted in PBS at pH 7.4 (1:10). Zetasizer Nano ZS (Malvern Instruments, Malvern, UK) was used to measure mean size and PdI, using Dynamic Light Scattering. All measurements were performed in triplicate.

- Determination of Conjugation Efficiency

To determine the conjugation efficiency (%) of the different HAOA-coated AuNPs functionalized formulations (i.e., EGF-functionalized, HTf-functionalized, and lapatinib-functionalized HAOA-coated AuNPs), the different formulations were centrifuged, at 7200× *g* for 15 min, as mentioned, and the supernatant was stored. Different methods were used to determine the concentration of ligand present in the formulations’ supernatant, and after this, concentration was determined, the conjugation efficiency was calculated using the following Equation (1):

(1) $$\begin{array}{cc} Conjugation\;Efficiency\;\left(\%\right) & =\frac{Initial\;Ligand\;Concentration-Ligand\;Concentration\;in\;the\;Supernatant}{Initial\;ligand\;Concentration}\times 100 \end{array}$$

(a) Determination of EGF concentration in the supernatant

To determine the concentration of EGF present in the supernatant of EGF-functionalized HAOA-coated AuNPs, after centrifugation, the following Bradford protocol was employed. Firstly, BSA solutions, with concentrations ranging from 0 to 25 µg/mL, were prepared in order to plot the calibration curve. Then, the sample/calibration curve solution were mixed with ThermoFisher Bradford Solution (ThermoFisher Scientific, Waltham, MA, USA) in a 96-well microplate, at a ratio of 1:1 (*v*/*v*), and left to incubate for 5 min. After this time period, the absorbance of the 96-well microplate was measured using a BioTekTM EL×800TM Absorbance Microplate Reader (Winooski, VT, USA), at 570 nm. The absorbance was then converted into concentration using Equation (2) plotted with the calibration curve solutions (R^2^ = 0.9801). This was then applied to Equation (1), allowing the determination of the conjugation efficiency. All measurements were performed in triplicate.

(2) $$Abs=\;0.0022\times EGF_{Concentration\;}+0.0331$$

(b) Determination of HTf concentration in the supernatant

The concentration of HTf present in the supernatant obtained from the centrifugation of HTf-functionalized HAOA-coated AuNPs was determined by using the A280 function in ThermoFisher NanoDropTM ND-1000 UV-Vis Spectrophotometer (ThermoFisher Scientific, Waltham, MA, USA). For this, a droplet (2 µL) of the supernatant was placed and the measurement was carried out. The obtained concentration was then applied to Equation (1) to determine the percentage of HTf that conjugated to the HAOA-coated AuNPs. All measurements were performed in triplicate.

(c) Determination of Lapatinib concentration in the supernatant

To determine the conjugation efficiency of lapatinib, the concentration of this ligand present in the supernatant was determined by HPLC (Hitachi System LaCrom Elite, Column oven, Diode Array Detector UV-vis and Pump, Tokyo, Japan), following a protocol described elsewhere [30]. The supernatants were analysed using a Column Water Symmetry C18 (5 µm, 4.6 × 150 mm) with isocratic flow of 0.5 mL/min, and a mobile phase composed of acetonitrile/water (70:30; *v*/*v*). Measurements were performed in triplicate at 227 nm, and a calibration curve was established using standardized solutions of lapatinib. The determined concentration was applied to Equation (1) in order to determine the conjugation efficiency of lapatinib to the surface of the HAOA-coated AuNPs.

- Morphology Analysis

The morphology of the different formulations, AuNPs’ core and HAOA-coated AuNPs, functionalized with different ligands and non-functionalized, was observed using Transmission Electron Microscopy (TEM). For TEM observations, aliquots (10 µL) of the aqueous suspensions of the different AuNPs formulations were carefully placed on 200-mesh copper grids previously coated with Formvar and Carbon and left to adsorb for 1–3 min. After removing the excess of the samples with filter paper, the material was negative staining with 1.0% of uranyl acetate for some minutes at room temperature, and further allowed to air-dry. Observations were made on a JEOL 1200EX transmission electron microscope (JEOL Ltd., Tokyo, Japan) operating at 80 kV. Images were recorded digitally.

#### 2.2.3. Determination of Absorbance Spectra of the HAOA-Coated AuNPs

The maximum absorbance wavelength for the AuNPs’ core, HAOA-coated AuNPs, and functionalized HAOA-coated AuNPs were determined by scanning the formulations’ absorbance at wavelengths ranging from 400 to 1000 nm, using a UV-Vis Spectrophotometer (Hitachi, Tokyo, Japan) against a blank. Formulations were diluted in PBS at pH 7.4 (1:4; *v*/*v*). The measurements were performed in triplicate.

#### 2.2.4. Determination of Recovery Yield

Prior to being recovered, all formulations (core, coated, and functionalized) were centrifuged (7200× *g*, 15 min) and freeze-dried using a FreeZone 2.5 Liter Benchtop Freeze Dry System (Labconco, Kansas City, MO, USA), at −49 °C for 48 h. After lyophilization, the powder was weighted and the recovery yield (RY, %) was calculated by using the following Equation (3):

(3) $$Recovery\;Yield{\;}_{\left(\%\right)}=\;\frac{Final\;mass\;of\;AuNPs}{Initial\;mass\;of\;all\;components\;used\;in\;formulation\;}\times 100$$

#### 2.2.5. In Vitro Safety Assessment

In vitro safety was assessed by evaluating cell viability (methylthiazolyldiphenyl-tetrazolium bromide, MTT, assay) of the non-cancerous cell line HaCat and the cancerous cell line 8505C after incubation with the different formulations when non-activated by laser. For this, HaCat cells and 8505C cells were separately seeded in a 96-well plate at a concentration of 7.5 × 10^4^ cells/well, and left to incubate with the formulations, for 24 h, at 20 µM and 80 µM of gold: core AuNPs, HAOA-coated AuNPs, EGF-functionalized HAOA-coated AuNPs, HTf-functionalized HAOA-coated AuNPs, lapatinib-functionalized HAOA-coated AuNPs, and the free ligands (EGF, HTf, and lapatinib). After 24 h, medium was removed, the cells were washed twice with PBS at pH 7.4, and 50 µL of MTT reagent (Sigma-Aldrich, St. Louis, MO, USA) was added at a concentration of 0.5 mg/mL (in incomplete DMEM). The cells were then left to incubate with the MTT reagent for 4 h, and then the formed crystals were solubilized using dimethyl sulfoxide (DMSO). Absorbance was measured using a BioTekTM EL×800TM Absorbance Microplate Reader (Fisher Scientific, NH, USA) at 570 nm. The control cells (incubated with complete medium) corresponded to 100% cell viability. In this study, the results are presented as percentage of the control cells and calculated according with Equation (4):

(4) $$Cell\;Viability\;\left(\%\right)=\;\frac{Abs_{test\;group}}{Abs_{Control}}\times 100$$

#### 2.2.6. In Vitro Efficacy Assessment

**Figure 1:**
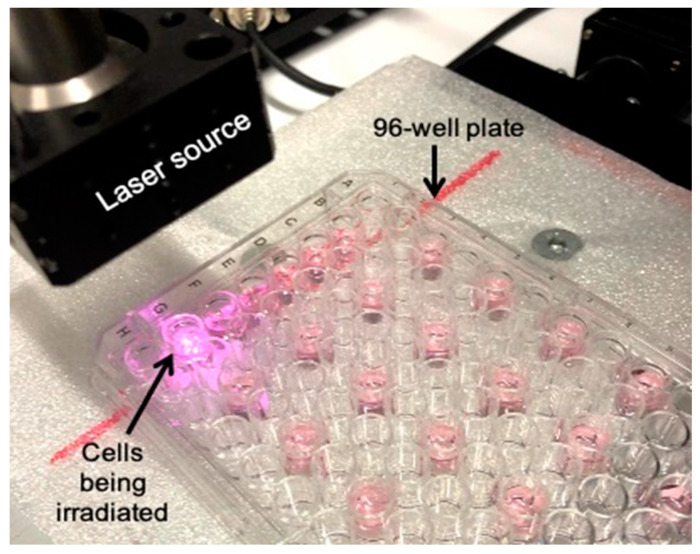
Laser setup for the in vitro activation of gold nanoparticles (AuNPs).

Efficacy was assessed in vitro by MTT assay. Human ATC cell line 8505C were seeded in two mirroring 96-well plate at a concentration of 7.5 × 10^4^ cells/well, and left to incubate with the formulations (AuNPs, HAOA-coated AuNPs, EGF-functionalized HAOA-coated AuNPs, HTf-functionalized HAOA-coated AuNPs, and lapatinib-functionalized HAOA-coated AuNPs), for 4 h, at 80 µM. After 4 h, medium was removed and replaced with fresh medium, and one of the plates containing the cells were irradiated with a laser (2495-L4, JDSU, CA, USA) emitting at a wavelength of 808 nm, 1.4 W, for 3 min, as shown in Figure 1, while the other plate was left without being irradiated. To evaluate the possible cytotoxicity of the laser, cells that were not incubated with formulations were also irradiated. The cells were washed twice with PBS at pH 7.4, and 50 µL of MTT reagent (Sigma-Aldrich, St. Louis, MO, USA) was added, at a concentration of 0.5 mg/mL (in incomplete medium). The cells were then left to incubate with the MTT reagent for 4 h in order to allow the crystals to form, posteriorly solubilized using DMSO. Absorbance was measured using a BioTekTM EL×800TM Absorbance Microplate Reader (Fisher Scientific, NH, USA) at 570 nm. Like the previous MTT assay, the control cells (incubated with complete medium) were correspondent to 100% cell viability. The results were also calculated using Equation (4) and presented as a percentage of controls.

#### 2.2.7. In Vitro Selectivity Assessment

Selectivity of the formulations was tested in vitro by determining the viability of 8505C cells after incubation with the different formulations for 24 h and, comparing to the results of the in vitro safety assessment using HaCat cells. For this, the cells were seeded in a 96-well plate at a concentration of 7.5 × 10^4^ cells/well, and left to incubate with the formulations (AuNPs, HAOA-coated AuNPs, EGF-functionalized HAOA-coated AuNPs, HTf-functionalized HAOA-coated AuNPs, and lapatinib-functionalized HAOA-coated AuNPs), for 24 h at 20 µM and 80 µM of gold. After this time period, the cells were washed twice with PBS at pH 7.4 and 50 µL of MTT reagent (Sigma-Aldrich, St. Louis, MO, USA) was added, at a concentration of 0.5 mg/mL (in incomplete medium). The cells were then left to incubate with the MTT reagent for 4 h and the crystals were solubilized using DMSO. Absorbance was measured using a BioTekTM EL×800TM Absorbance Microplate Reader (Fisher Scientific, NH, USA), at 570 nm. In similarity to the previous MTT assays, the control cells (incubated with complete medium) corresponded to 100% cell viability, and the results, in percentage, were calculated using Equation (4).

#### 2.2.8. In Vitro Haemolytic Activity

The used protocol was based on a previously published one [31]. Briefly, ethylenediamine tetraacetic acid (EDTA)-preserved peripheral human blood was collected from voluntary donors at the day of the determination of the haemolytic activity of AuNPs’ core, HAOA-coated AuNPs, and HTf-functionalized HAOA-coated AuNPs. The peripheral blood was centrifuged at 1000× *g* for 10 min to separate and remove the plasma from the erythrocytes. After this, the erythrocytes were suspended in PBS and centrifuged at 1000× *g* for 10 min, three times. The formulations were diluted in PBS to concentrations ranging from 0.04 to 80 µM (2-fold dilution). One hundred μL of the diluted formulations were distributed into 96-well plates and then 100 μL of the erythrocyte suspension was added to each well. Erythrocytes and distilled water (equal volumes) were added to 6 wells, corresponding to 100% haemolysis (positive control) and erythrocytes and PBS were added to other 6 wells, corresponding to 0% haemolysis (negative control). Plates were left to incubate for 1 h at 37 °C and then centrifuged at 800× *g* for 10 min. The supernatants were collected and the absorbance (Abs) was measured using a BioTekTM EL×800TM Absorbance Microplate Reader (Winooski, VT, USA) at 570 nm with a reference filter of 620 nm. The haemolytic activity (%) was determined according to the following Equation (5):

(5) $$Haemolytic\;Activity\;\left(\%\right)=\;\frac{Sample\;Abs-Negative\;Control\;Abs}{Positive\;Control\;Abs-Negative\;Control\;Abs}\times 100$$

#### 2.2.9. Preliminary in Vivo Assessment of HTf-Functionalized and Non-Functionalized HAOA-Coated AuNPs

- In Vivo Preliminary Safety Assessment

An in vivo preliminary safety assessment of the HAOA-coated AuNPs was performed using 18 CD1 mice with a mean average body weight of 23 g. The formulation was administered subcutaneously in the thyroid neck region, at a dosage of 23 mg/kg of body weight. Then, the animals were randomly separated into six groups, each with 3 specimens, being sacrificed at different time points: 30 min (group 1), 1 h (group 2), 2 h (group 3), 4 h (group 4), 6 h (group 5), and 24 h (group 6). At each time point, the animals of each group were anesthetized and sacrificed by cervical dislocation, according to animal welfare principles. Afterwards, peripheral blood samples were collected for biochemical analysis, pictures of the neck region were taken and organs and tissues (i.e., neck region including thyroid, spleen, right lobe of the liver, right kidney, skin from administration site) were harvested for histological examination. For biochemical analysis, plasma was separated from whole blood. In order to do this, the collected blood was centrifuged at 1000× *g* for 10 min and the supernatant, corresponding to plasma, was collected for quantification of IL-6, Alanine Aminotransferase (ALT, to determine liver toxicity), creatine, and urea (to determine kidney toxicity).

For histological analysis, samples were fixed in 10% formalin and processed for routine H&E staining. Histopathological assessment was performed using an Olympus CX51 microscope (Olympus Corporation, Tokyo, Japan). Whole slide scanning was performed using the NanoZoomer-SQ Digital slide scanner—C13140-01 (Hamamtsu, Japan) and representative photos were taken using the NDP.View2 software.

- In Vivo Biodistribution Assessment of HTf-Functionalized HAOA-Coated AuNPs

The biodistribution studies of HTf-functionalized HAOA-coated AuNPs and HAOA-coated AuNPs were assessed using 12 CD-1 mice. The animals were randomly distributed in different groups and administered either HTf-functionalized HAOA-coated AuNPs or HAOA-coated AuNPs. In similarity to the previous assay, the respective formulations were administered s.c. in the thyroid region at a dose of 23 mg/kg of body weight. The animals of each group were then re-divided into two subgroups, according to the time point at which they would be sacrificed: 4 h and 24 h post-administration. Respecting the animal welfare principles, the animals were sacrificed and the main organs involved in clearance and excretion (i.e., liver, spleen, kidneys, and lungs) were harvested and weighed to determine its’ tissue indexes, according to the following Equation (6) [32]:

(6) $$Tissue\;Index=\;\sqrt{\frac{organ\;weight}{animal\;weight}}\times 100$$

After weighing the organs, Inductively Coupled Plasma-Mass Spectrometry (ICP-MS) was conducted to determine the amount of Hf-functionalized HAOA-coated AuNPs and HAOA-coated AuNPs present in each of these organs (i.e., liver, spleen, kidneys, and lungs) as well as in the blood. For this analysis, the organs and blood were freeze dried and stored at −80 °C and further digested using a microwave digestive system and a mixture of nitric acid, hydrochloric acid, and hydrogen peroxide.

#### 2.2.10. Statistical Analysis

Data is presented as mean ± SD, with a n ≥ 3. GraphPad Prism v5.03 (GraphPad Software, San Diego, CA, USA) was used to perform all the statistical analysis. Statistical differences were evaluated with ANOVA, and the differences were considered as significant for *p* < 0.05.

## 3. Results

### 3.1. Development and Characterization of the Functionalized HAOA-Coated AuNPs

#### 3.1.1. Mean Size, Polydispersivity Index (PdI), and Recovery Yield (RY)

**Figure 2:**
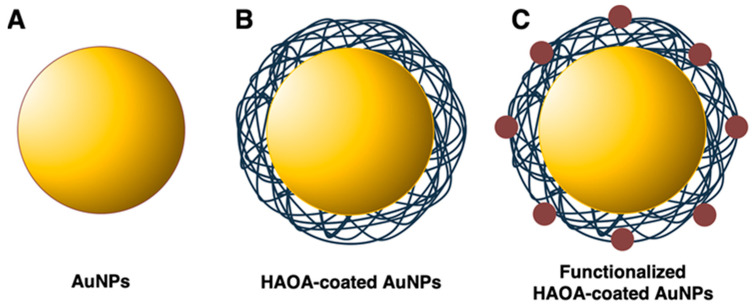
Gold nanoparticles (AuNPs) throughout the preparation steps: (**A**) AuNPs’ core; (**B**) HAOA-coated AuNPs; and (**C**) functionalized (i.e., EGF, HTf, or lapatinib) HAOA-coated AuNPs.

AuNPs were prepared using two different ratios of gold:RA, 5:3 and 5:10 (m/m). The AuNP core (Figure 2A) was formulated following the presented ratios, and the NPs were coated with the HAOA coating (Figure 2B). Mean size and PdI were determined throughout the different steps of assembling the final formulation (i.e., core synthesis, coating of the NPs, and functionalization of the AuNPs’ surface). The main results are presented in Table 1. Particle size significantly varied when different gold:RA ratios (m/m) were used and tended to increase when the HAOA coating was added, for both AuNPs using 5:3 (increase of ~40%) and 5:10 (increase of ~12%) (m/m) gold:RA ratios, suggesting that the AuNP’s core was successfully coated. Concerning all previous results, the 5:10 AuNPs core was then chosen for functionalization, using three different ligands, EGF, HTf, and Lap (Figure 2C). After functionalization, mean size and PdI were determined once more, and it is noted that there was a tendency of increase in size, significant in the case of the EGF-functionalized HAOA-coated AuNP.

Moreover, the RYs of the NPs were determined for the uncoated core, coated, and functionalized formulations. The values ranged from 58 to 86%. HAOA-coated and EGF-functionalized HAOA-coated 5:10 AuNPs presented the lowest RYs, while lapatinib-functionalized HAOA-coated 5:10 AuNPs displayed the highest RYs, achieving a mean value of 86%.

#### 3.1.2. Morphological Analysis

**Figure 3:**
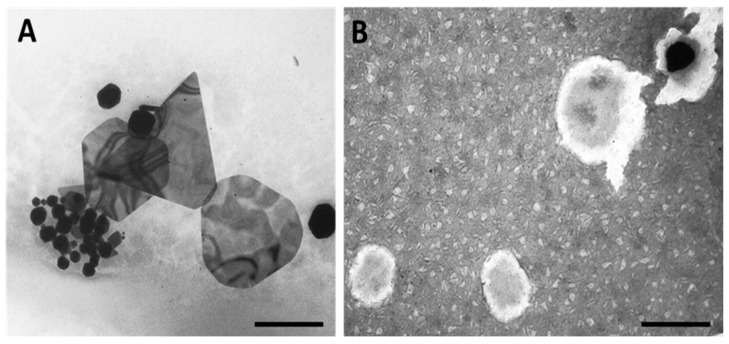
TEM micrographs of 5:3 gold:RA (m/m) AuNPs: (**A**) uncoated and (**B**) HAOA-coated. Scale bars = 200 nm.

**Figure 4:**
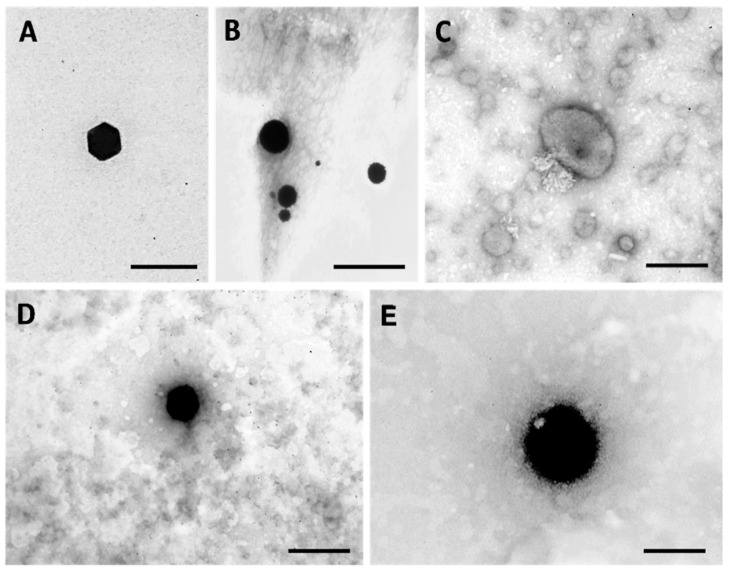
TEM micrographs of ratio 5:10 gold:RA (m/m) AuNPs: (**A**) uncoated; (**B**) HAOA-coated; (**C**) Lap-functionalized HAOA-coated; (**D**) HTf-functionalized HAOA-coated; (**E**) EGF-functionalized HAOA-coated. Scale bars = 100 nm (**A**); 200 nm (**B**–**E**).

Morphology of the AuNPs was assessed by Transmission Electron Microscopy (TEM). Representative images are shown in Figure 3 for 5:3 gold:RA (m/m) uncoated and HAOA-coated, while Figure 4 exhibits representative images of 5:10 gold:RA (m/m) ratio AuNPs, uncoated, HAOA-coated, and functionalized using the three ligands (EGF, HTf, and lapatinib).

The 5:3 AuNPs have in general a spherical or a polyhedral shape with different sizes (Figure 3A). When coated with HAOA, the 5:3 AuNPs show a black AuNP core surrounded by a cloudy coating, probably related to the HAOA mixture (Figure 3B).

Although spherical particles were also present, 5:10 AuNPs were predominantly polyhedral shaped. In Figure 4A, a very well-defined hexagonal face of a polyhedral shaped-uncoated 5:10 AuNPs can be clearly observed. As previously seen for the 5:3 AuNPs (Figure 3B), the shape of the 5:10 AuNPs (Figure 4A) also changed after HAOA coating addition (Figure 4B). Lap-functionalized HAOA-coated AuNPs (Figure 4C), HTf-functionalized HAOA-coated AuNPs (Figure 4D), and EGF-functionalized HAOA-coated AuNPs (Figure 4E) showed an evident size increase in comparison with HAOA-coated NPs (Figure 4B), a fact that is confirmed by size analysis.

#### 3.1.3. SPR Band

Aiming to achieve more effective AuNP-mediated PTT, AuNPs must absorb at wavelengths within the biological windows (650–1400 nm). Thus, the main goal was to develop functionalized HAOA-coated AuNPs that absorbed in the NIR range (650–950 nm) of the light spectrum, as these wavelengths are described to safely penetrate healthy tissues and to allow the activation of AuNPs localized in deeper tumours [19], such as thyroid malignancies. To evaluate this, the SPR band, corresponding to the maximum absorbance peak (Abs_max_) of the AuNPs, was determined throughout the different steps of development, for both gold:RA ratios used (Table 2). It is worth noticing that while uncoated 5:10 AuNPs presented a SPR band in the NIR wavelengths, such results were not observed for the 5:3 uncoated AuNPs. It would be expected to shift to higher wavelengths when the AuNPs were coated with HAOA. Although this was the case for the HAOA-coated 5:10 AuNPs, with a shift from 753 ± 54 to 840 ± 122 nm, a SPR band decrease from 551 ± 26 nm to 536 ± 2 nm was observed for the 5:3 AuNPs uncoated and coated with HAOA, remaining in the visible range. Due to this, the 5:10 AuNPs were chosen for the next stage of the formulation design, functionalization with the ligands. It is to note that after functionalization with the different ligands, the SPR bands of the EGF- and HTf-functionalized HAOA-coated 5:10 AuNPs remained in the NIR range (785 ± 127 and 710 ± 128 nm, respectively), but the Abs_max_ lapatinib-functionalized HAOA-coated 5:10 AuNPs (481 ± 25 nm) shifted into the visible section of the light spectrum.

#### 3.1.4. Conjugation Efficiency

Due to the favourable characteristics of the 5:10 AuNP core (i.e., SPR band in the NIR range) when compared to the 5:3 AuNP, the 5:10 AuNP formulation was chosen for functionalization and further testing, being from now referred as AuNPs. Conjugation efficiency was indirectly determined by measuring the non-conjugated free protein present in the supernatant after centrifugation by colorimetric assays or by protein quantification, regarding EGF and HTf, respectively. Results are summarized in Table 3. HTf-functionalized HAOA-coated AuNPs had a higher conjugation efficiency than the EGF-functionalized HAOA-coated AuNPs. The quantification of the non-conjugated free lapatinib was performed by HPLC.

### 3.2. Preliminary Safety Assessment

**Figure 5:**
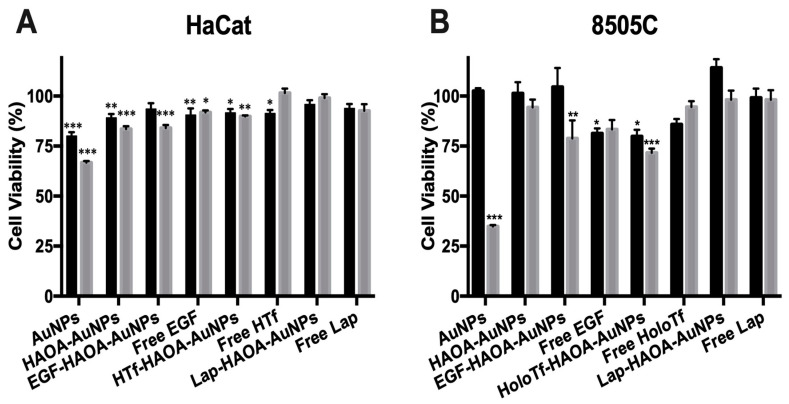
Evaluation of HaCat (**A**) and 8505C (**B**) cell viability, after a 24 h incubation period with the different AuNPs formulations (coated and uncoated, and functionalized with the different ligands and non-functionalized) at two different concentrations (20 µM, in black columns and 80 µM, in grey columns). Results are shown regarding mean ± SD, *n* = 5 (* *p* < 0.05, ** *p* < 0.01, *** *p* < 0.001 vs. control).

A preliminary in vitro safety evaluation of the different formulations was performed for healthy tissues using HaCat cells, a human epithelial cell line, and for tumours using 8505C cells, a human ATC cell line. Formulations were incubated for 24 h, at two different concentrations (20 and 80 µM). The selected concentrations were based on our previous studies [27,28]. The results of this in vitro assay are presented in Figure 5A for HaCat and in Figure 5B for 8505C. There was a slight decrease in cell viability for some of the tested formulations when compared to cells in the presence of complete medium (100% viability). Cell viability of HaCat cells was higher than 70% for all tested formulations and concentrations, thus demonstrating their safety according to ISO 10993-5:2009(E) [33]. Regardless, addition of the HAOA coating seems to have improved cell viability when compared to the uncoated-AuNPs for both concentrations, increasing cell viability of HaCat cells by ~9% and ~17%, at 20 and 80 µM, respectively, and increasing cell viability of 8505C cells by ~60%, at 80 µM. Moreover, all the functionalized (EGF, HTf and lapatinib) HAOA-coated AuNPs did not decrease cell viability below 85%, and thus seem to be safe for in vitro non-pathologic tissues.

### 3.3. In Vitro Efficacy Assessment

**Figure 6:**
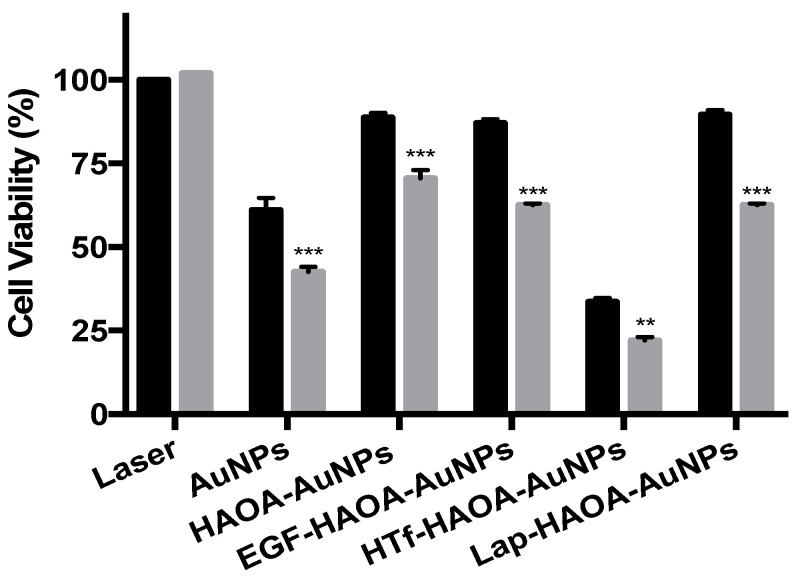
Evaluation of 8505C cells viability after a 4 h incubation period with different AuNPs formulations at 80 µM, activated with 3 A (grey columns) or not laser activated (black columns). Results are shown regarding mean ± SD, *n* = 3 (** *p* < 0.01, *** *p* < 0.001 vs. control).

The efficacy of the formulations against ATC was assessed using 8505C cells, a human ATC cell line. For this, cells were incubated with the formulations under study at 80 µM for 4 h, thus allowing the formulations to target the cells, bind cellular receptors, and be internalized by them. To activate the formulations, the cells were then irradiated with a laser, emitting at 808 nm, with 3 A for 3 min, and cell viability was then determined. The 8505c cells were also incubated with the same formulations, non-activated, to compare the activity of formulations after activation. Moreover, to ensure that differences in cell viability observed were not caused by the laser, a group of cells were incubated with complete medium and then were irradiated, enabling to evaluate the solo effect of the laser in cell viability. The cell viability results regarding the formulation efficacy are shown in Figure 6. All laser-activated samples presented caused a significant decrease in 8505C cell viability when compared to the respective non-activated formulations. Furthermore, it was clear that the HTf-functionalized HAOA-coated AuNPs were the most cytotoxic for the ATC cell line, both for activated and not activated. Moreover, for cells incubated with complete medium, the laser did not induce any effect on cell viability.

### 3.4. In Vitro Selectivity Assessment

**Figure 7:**
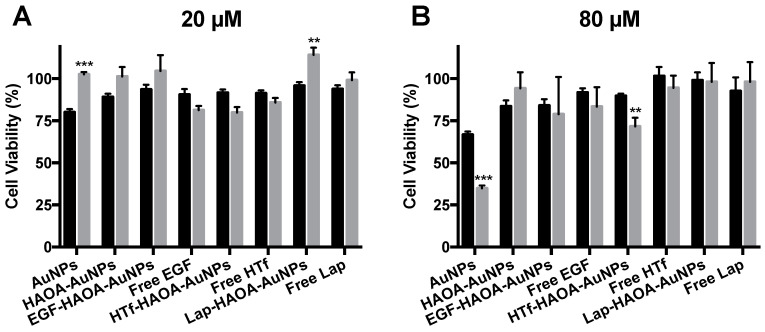
Evaluation of HaCat (black columns) and 8505C (grey columns) cell viability after a 24 h incubation period with the non-activated AuNPs formulations: (**A**) 20 and (**B**) 80 µM. Results are shown regarding mean ± SD, *n* = 5 (** *p* < 0.01, *** *p* < 0.001 vs. control).

To complete the in vitro testing, the different AuNPs formulations’ selectivity was assessed by incubating HaCat and 8505C cells with the formulations under study at several concentrations (20 and 80 µM) for 24 h. Results are shown in Figure 7. By comparing the viability of both cell lines incubated for 24h, with formulations at 20 µM (Figure 7A), it was observed that some formulations (i.e., AuNPs, HAOA-coated AuNPs, EGF-functionalized HAOA-coated AuNPs and lapatinib-functionalized HAOA-coated AuNPs) were more cytotoxic for the HaCat cells than for the cancerous 8505C cells. In fact, the HTf-functionalized HAOA-coated AuNPs at 20 µM were the only formulation to which the 8505C were more susceptible, presenting a cell viability of 79.83 ± 3.36%, while for HaCat cells a cell viability of 91.67 ± 1.87% was achieved. The results were slightly different when the formulations tested were at a concentration of 80 µM (Figure 7B). AuNPs and HTf-functionalized HAOA-coated AuNPs resulted in significantly different cell viability results depending on the cell line, showing some selectivity for the ATC cell line, as demonstrated by a more pronounced reduction in cell viability to the 8505c cells (34.83 ± 0.70% and 71.67 ± 2.06%, for each respective formulation) in comparison to data observed for HaCat cells (66.80 ± 0.80% and 89.83 ± 0.54%, respectively).

Based on these and the previously presented in vitro results, HTf-functionalized HAOA-coated AuNPs were the formulation selected for further experiments.

### 3.5. In Vitro Haemolytic Activity

**Figure 8:**
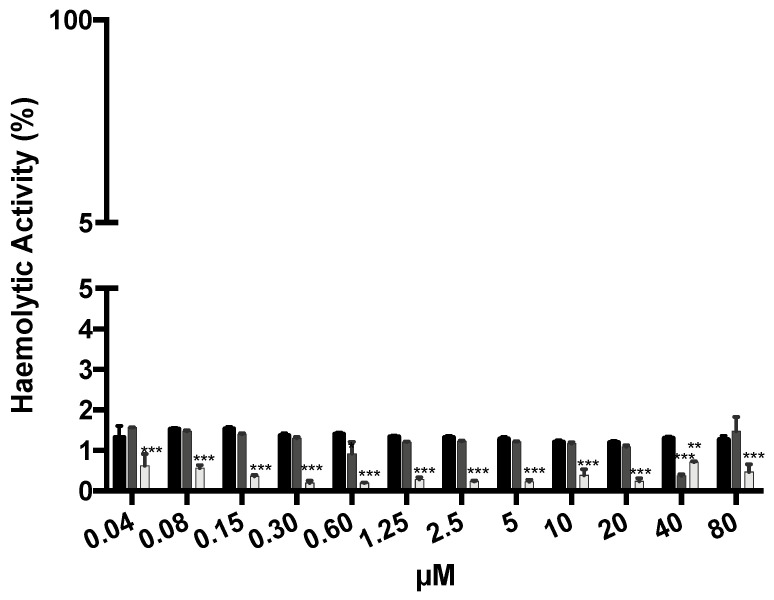
Haemolytic activity of AuNPs (black columns), HAOA-coated AuNPs (dark grey columns), and HTf-functionalized HAOA-coated AuNPs (light grey columns) at concentrations ranging from 0.04 to 80 µM. Results are shown regarding mean ± SD, *n* = 3 (** *p* < 0.01, *** *p* < 0.001 vs. control).

The haemolytic activity of the formulations was determined at a wide range of concentrations. The results are summarized in Figure 8. For all concentrations tested, haemolytic activity was below 2%, which is a good indicator for the safety and biocompatibility of all formulations, an important aspect to consider when dosage formulations are intended for parenteral administration in future studies.

### 3.6. In Vivo Safety Assessment

**Figure 9:**
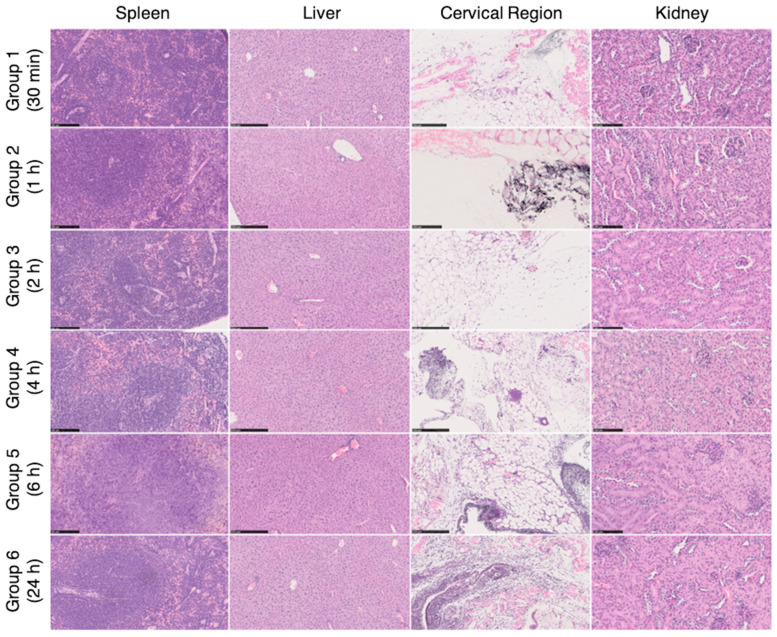
Histological images of the spleen, liver, cervical region and kidney removed for histopathological analysis after necropsy (spleen and kidney with 200× magnification; liver and neck area with 100× magnification). On the cervical region, in the subcutaneous tissue, a mixed inflammatory infiltrate was observed, composed mainly of neutrophils and macrophages, associated with a black granular pigment. Some cells had this pigment on the cytoplasm. These findings showed higher grades of severity on group 5 and 6 (6 h and 24 h, respectively). For the remaining organs, no histological alterations were observed (H&E staining).

Ex vivo histopathological analysis of different organs (spleen, liver, cervical region—administration site, and kidney) were performed at different time points (0.5, 1, 2, 4, 6, and 24 h) following the subcutaneous tissue, adjacent to the administration of HAOA-coated AuNPs at the cervical region of mice. Representative images are shown in Figure 9. In most of the groups, an accumulation of the HAOA-coated AuNPs (seen as a black-ish granular material) was observed in the s.c. adipocyte tissue, adjacent to the administration site. HAOA-coated AuNPs induced an inflammatory reaction with the presence of cellular infiltrates, more pronounced at 24 h post-administration, mainly composed of macrophages and neutrophils. The cytoplasm of these inflammatory cells was filled with dark granular material, compatible with HAOA-coated AuNPs. Regarding the other organs, no abnormalities were seen.

### 3.7. Preliminary In Vivo Biodistribution Assessment

The tissue indexes of the main excretion organs (i.e., liver, spleen, kidneys, and lungs) were determined and the results are shown in Table 4. HAOA-coated AuNPs increased significantly the tissue index of the liver at 24 h post-administration. However, the administration of HAOA-coated AuNPs did not result in changes on the respective tissue indexes of the organs under study. HTf-functionalized HAOA-coated AuNPs led to a significant increase of the tissue index of the liver at both 4 h and 24 h post-administration, but this increase was more significant at 24 h. As for the non-functionalized formulation, HTf-functionalized HAOA-coated AuNPs did not trigger any other significant variations of the spleen, kidneys, and lungs’ tissue indexes.

**Figure 10:**
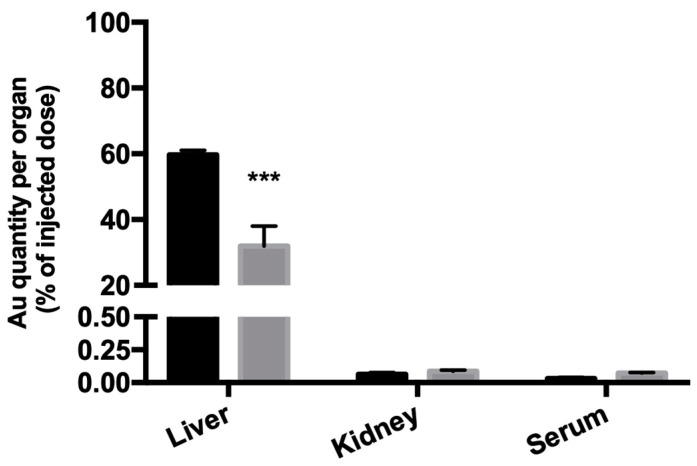
Gold content (ICP-MS) in the liver, kidney, and serum of mice 24 h post-s.c. administration of HAOA-coated (dark grey columns) and HTf-functionalized HAOA-coated AuNPs (light grey columns). Results are presented as mean ± SD, *n* ≥ 3 (*** *p* < 0.001 vs. HAOA-coated AuNPs).

The gold content at 24 h post-s.c. administration of the formulations tested (HAOA-coated AuNPs and HTf-functionalized HAOA-coated AuNPs), in relation (%) to the injected dose of gold (23 mg of formulation/kg body weight) of the liver, kidneys, and serum was determined by ICP-MS, and the results are shown in Figure 10. The two formulations poorly accumulated in the kidney and serum, as intended, with residual percentages of gold (less than 0.25%), with the functionalization resulting in any significant differences regarding gold content. However, functionalizing the HAOA-coated AuNPs with HTF resulted in a significant decrease in the gold content in the liver: from 60% of the injected dose when not functionalized (HAOA-coated AuNPs) to 30% of the injected dose when functionalized (HTf-functionalized HAOA-coated AuNPs).

## 4. Discussion

Several methods are described for the preparation of AuNPs, such as the Turkevich method, Seeding Growth method, chemical method, the Brust-Schiffrin method, e.g., [34]. However, the majority of those methods uses cetyltrimethil ammonium bromide (CTAB), which is an inflammable and cytotoxic agent that raises important safety concerns [26,35]. Therefore, there was the need to develop CTAB-free AuNPs, and the method described in the present work was able to successfully overcome this challenge, as no CTAB was used. In this study, the method used to produce AuNPs was adapted from the one previously developed by our group for the treatment of melanoma [27,28]. Our previous work included plant extract, which led to some problems in terms of reproducibility. In the present study, for the AuNPs preparation, rosmarinic acid was used instead of the plant extract. All the other compounds used for the AuNPs preparation are safe for clinical application [36,37,38,39,40]. Moreover, the ratio between rosmarinic acid and gold was optimized.

These new AuNPs were characterized in each step of production (i.e., core synthesis, coating addition, and functionalization of the AuNPs’ surface) regarding its size, PdI, SPR band correspondent to the maximum absorbance peak, recovery yield (RY), surface morphology, and conjugation efficiency.

When using a 5:3 gold:RA ratio (m/m) for the synthesis of AuNPs, the resultant core mean size was 88 nm, while using a 5:10 gold:RA ratio (m/m) resulted in AuNPs cores with much higher mean size (~315 nm; *p* < 0.001). When the AuNPs were coated with HAOA, particle size increased. It is notable that this tendency of increasing mean size after coating was more pronounced for the formulation synthetized using the 5:3 gold:RA ratio (~40%) in comparison to the 5:10 gold:RA ratio (~12%). The formulations herein described are intended to be administered in situ and to remain in the target area. Thus, formulation clearance (both reticuloendothelial and hepatic cells) should occur only after activation of the formulations. This clearance rate is NPs’ size-dependent, since it has been described that phagocytic cells are able to uptake NPs with mean sizes up to 1 µm [41,42]. On the other hand, as the formulation should not enter into the systemic circulation, thus, NPs size should be greater than 200 nm [41,42]. Having these size-related clearance aspects in consideration, the 5:10 AuNP core seems to be more adequate than both the 5:3 AuNP and previously designed formulation for melanoma [27,28]. In addition, since these cores are smaller and, even after coating with HAOA, remain smaller than 200 nm, they represent a higher risk of entering into the systemic circulation by intravasion through the tumour leaky vasculature [41,42].

Still regarding the characterization of the AuNPs size, PdI of the 5:3 and 5:10 AuNPs was determined, allowing to assess homogeneity of the developed formulations. More uninform AuNPs were observed with the 5:3 ratio, uncoated and coated. PdIs up to 0.5 are deemed acceptable, according to literature [43]. Moreover, although the 5:10 AuNPs core PdI was higher (0.475 ± 0.029), it decreased following HAOA coating (0.394 ± 0.176), which suggests that the coating contributes for uniformization of the 5:10 AuNPs size. Indeed, it is important for a formulation to be monodisperse, as this characteristic uniformizes the formulations properties, as well as it allows great predictability for the in vitro and in vivo NPs behaviour [44,45,46].

AuNPs size also seems to influence the SPR band/Abs_max_ of AuNPs. In fact, as AuNPs size increases, the SPR band seems to move towards the right end of the radiation spectrum to higher wavelengths [47]. Moreover, SPR band is also influenced by PdI of the AuNPs, with more uniform populations of AuNPs having a more defined peak of absorbance for a certain wavelength, unlike populations with higher PdIs, which absorb throughout a broader range of wavelengths [48]. This was the case of the results presented herein, with the smaller AuNPs (5:3) having a SPR band in the visible region of the light spectrum, and the larger AuNPs (5:10) having a SPR band in NIR wavelengths. The HAOA coating, used to increase biocompatibility (discussed below), is also expected to cause a shift on the absorbance towards higher wavelengths, i.e., to the right end of the light spectrum. This was reflected in the 5:10 AuNPs, as the SPR band shifted to 840 nm with the coating addition, whereas in the 5:3 AuNPs SPR band remained unchanged (see Table 2). Taking into consideration that it is of interest that the final formulation of AuNPs would absorb in the wavelength range of the previously mentioned biological window, the SPR band presented by the 5:10 AuNPs seems to be more adequate. Furthermore, 5:10 AuNPs present an improved higher SPR band (840 nm) when compared to the previously reported formulation developed for melanoma (709 nm) [27]. This represents a significant improvement, as the laser used for the activation of AuNPs emits at 808 nm.

In order to achieve fully functional AuNPs, selective for ATC, ligand(s) were chosen for surface modification of the formulation. The formulation’s targets were chosen by choosing membrane receptors known to be overexpressed in ATC cells. Two target receptors were selected: epidermal growth factor receptor (EGFR) [49,50,51,52] and type I receptor of transferrin (TfR1/CD71) [53,54,55]. In order to target those receptors, three ligands were selected for the surface functionalization: human EGF, the ligand for EGFR; lapatinib, an EGFR and HER-2 tyrosine kinase inhibitor [56]; human Holo-Transferrin (HTf), the iron-bound form of the ligand (transferrin) for TfR1/CD71.

Formulations were characterized regarding its recovery yield (RY) and surface morphology, and the efficiency of surface functionalization was performed by indirectly determining the amount of ligand at NPs’ surface. Surface morphology, assessed by TEM, showed that the 5:10 AuNPs core has a very well-defined polyhedral shape. In fact, the 5:10 AuNP core is much more uniform than the previously described formulation, which was heterogenous [27] and more similar to the 5:3 AuNPs’ core. Regarding the functionalization of the HAOA-coated 5:10 AuNPs, there was no difference in shape before (HAOA-coated AuNPs) and after functionalization with either EGF or HTf, as the shape remained spherical and well defined. However, the shape changed after lapatinib functionalization, with both spherical and more elongated lapatinib-functionalized HAOA-coated 5:10 AuNPs. The functionalization also induced a change in the HAOA-coated AuNPs size: the coated AuNPs size increased two-fold when functionalized with EGF, in contrast to 1.1 to 1.4-fold when functionalized with HTf and lapatinib. Nevertheless, all functionalized formulations displayed mean sizes that met the criteria for ideal NPs as previously discussed, i.e., between 200 nm and 1 µm.

The efficiency of ligand conjugation to the HAOA-coated AuNPs was determined for EGF, HTf, and lapatinib. All ligands conjugated efficiently (>70%) at the surface of the HAOA-coated AuNPs, with EGF having the lowest values. RY indicates the percentage of materials/reagents that formed AuNPs and is a very important parameter for industrial scale level. All formulations presented an adequate RY, with 66.0 ± 0.9% for EGF, 70.0 ± 3.4% for HTf, and 86.2 ± 36.5% for lapatinib.

The relationship between AuNPs SPR band and how both HAOA coating and ligand-binding affects absorbance of the functionalized coated AuNPs is also important for formulation efficacy and, therefore, matter for discussion. Since the ligands EGF, HTf, and lapatinib present maximum absorption at wavelengths in the UV range, the SPR band of the functionalized formulations shifted towards the left end of the light spectrum when compared to the non-functionalized HAOA-coated AuNPs. Moreover, functionalizing the HAOA-coated AuNPs with lapatinib caused a more pronounced shift, culminating in a SPR band in the visible range of the light spectrum. As mentioned, it is of interest to formulate AuNPs that absorb NIR wavelengths (650–950 nm), favouring the 5:10 formulation. Due to the 5:10 ratio gold:RA (m/m), AuNPs having advantageous size and SPR band, this was the selected formulation that was functionalized and used in the further assays.

Two of the biggest concerns during the formulation development is its safety and efficacy. The formulation must be biocompatible with healthy tissues, and cytotoxic and selective for ATC cells, upon activation by laser. For this purpose, safety, efficacy, and selectivity were studied in vitro using two cell lines: HaCat, a human healthy keratinocyte cell line, and 8505C, a human ATC cell line. Safety was assessed by incubating HaCat and 8505C cells with different concentrations of the several formulations for 24 h. As expected, the uncoated AuNPs at a concentration of 80 µM were cytotoxic for the healthy (and for the cancerous) cells, which was reduced by coating them with HAOA, rendering the coated AuNPs nontoxic, i.e., biocompatible. According to ISO 10993-5:2009(E), formulations that result in a decrease of cellular viability greater than 30% are classified as cytotoxic [33]. Thus, according to ISO standards, all coated formulations tested, including both functionalized and non-functionalized, were biocompatible with HaCat cells at 20 and 80 µM concentration, as the maximum decrease in cell viability observed was only 17% in comparison to the control. As for HaCat, the AuNPs were cytotoxic for 8505C at 80 µM, which was also resolved by coating the AuNPs with HAOA. Moreover, the cell viability of 8505C was overall lower than HaCat cell viability for the different formulations at 80 µM, but the selectivity of the formulations will be addressed bellow.

The efficacy of the different formulations was studied by analysing cellular viability after incubation with the laser-activated and non-activated AuNPs, HAOA-coated AuNPs, and functionalized HAOA-coated AuNPs. The cytotoxicity action of the laser was also addressed by irradiating non-treated cells with the laser used to activate the formulations, and no reduction in cell viability was observed. Furthermore, the safety of this methodology has been previously assessed by our group using a similar formulation in HaCat and yeast models [29]. In addition to its in vitro safety, the laser system used to activate the formulations has shown to be very suitable and easily implementable for clinical applications, mainly due to its easy and intuitive manipulation, but also due to the device mobility. Regarding the formulations, it is to note that, when non-activated, uncoated-AuNPs and HTf-functionalized HAOA-coated AuNPs were effective in reducing ATC cells viability to 61% and 34%, respectively. However, the cell viability was greatly reduced to 42% (AuNPs) and 22% (HTf-functionalized HAOA-coated AuNPs), when a laser was used to activate the formulations. Thus, laser-induced activation of the formulations resulted in a significant decrease in cell viability when compared with non-activated formulation. According to ISO 10993-5:2009(E), the non-activated HAOA-coated AuNPs, EGF-functionalized HAOA-coated AuNPs and lapatinib-coated HAOA-coated AuNPs were not cytotoxic for ATC cells, resulting in cell viabilities of 89%, 87%, and 77%, respectively. Although activating HAOA-coated AuNPs and EGF-functionalized HAOA-coated AuNPs resulted in significant cell viability decrease, when compared with the respective non-activated formulations, HAOA-coated AuNPs still had no cytotoxicity against the 8505C cells, according to ISO standards. Despite the fact that EGF- and Lapatinib-functionalized HAOA-coated AuNPs are cytotoxic against ATC cells, both decreasing cell viability to 62% when activated, HTf-functionalized HAOA-coated AuNPs was the most effective formulation, with the highest toxicity against ATC cells, when activated (cell viability of ~22%) and for non-activated (cell viability of ~34%) at 80 µM. Therefore, data suggests that surface functionalization of the HAOA-coated AuNPs is crucial to increase the cytotoxic action against the ATC cell line, being HTf-functionalized HAOA-coated AuNPs the most effective formulation.

Selectivity was assessed by comparing cell viabilities of HaCat cells (non-pathological cells) and 8505C (ATC cells), after being incubated for 24 h with the different formulations, non-activated by laser, at 20 and 80 µM. The formulation was considered to be selective for ATC, as the cell viability observed for 8505C was significantly lower in comparison to HaCat cells, for the highest concentration tested (80 µM). At 80 µM, two formulations significantly decreased cell viability: AuNPs; and HTf-functionalized HAOA-coated AuNPs. Although AuNPs seems to be selective and cytotoxic for ATC cells, it failed to be biocompatible for healthy cells (HaCat), and thus, HTf-functionalized HAOA-coated AuNPs seem to be the most suitable formulation. This formulation was biocompatible with HaCat cells, demonstrating a very small decrease in viability of this cell line, and it was also the most effective against ATC cells, both when activated and when not activated by laser. Thus, our data suggest that HTf-functionalized HAOA-coated AuNPs is selective for the ATC cells and, therefore, this was the selected formulation for the in vivo experiments that aimed to assess safety and biodistribution, discussed below.

Although the final formulation is intended for intratumoural or in situ administration, there is still the low possibility that it could reach the bloodstream through its clearance or by intravasion of tumour vessels. Thus, the haemolytic activity was evaluated to ensure that formulations (AuNPs, HAOA-coated AuNPs, and HTf-functionalized HAOA-coated AuNPs) were safe for erythrocytes. For this, a wide range of concentrations of the formulations under study were incubated with red blood cells (0.04 to 80 µM). The obtained results demonstrated the safety of all formulations that presented an haemolytic activity below 2%. According to literature, formulations with less than 10% are considered non-haemolytic [57]. Therefore, one can state that AuNPs, HAOA-coated AuNPs, and HTf-functionalized HAOA-coated AuNPs formulations are safe parenteral administration and/or in case they reach the bloodstream or their cellular components, namely erythrocytes.

Biodistribution and safety assessments were separately carried out in vivo using HAOA-coated AuNPs and HTf-functionalized HAOA-coated AuNPs. For the safety assessment, HAOA-coated AuNPs were administrated s.c. at thyroid neck region of mice. At most timepoints tested, the HAOA-coated AuNPs were still present in the s.c. adipocyte tissue, adjacent to the administration site. In fact, HAOA-coated AuNPs seem to trigger the presence of macrophages at around 24 h, with cellular infiltrates with phagocyted AuNPs being observed. It has been described that although AuNPs are rapidly phagocyted after administration, polymeric coatings are associated with reduced phagocytosis [58]. Furthermore, when phagocyted or taken-up by human cells, AuNPs do not seem to be cytotoxic [59], as was also observed in the HaCat in vitro viability assays. This could explain why these infiltrates were only present at 24 h, as the functionalization and coating are expected to dramatically change the biodistribution of the AuNPs in comparison to non-functionalized AuNPs formulations. Moreover, no changes in the analysed organs were observed indicating that the absence of AuNPs-induced lesions and/or rapid clearance. Indeed, clearance was not significant before the 24 h timepoint, which is of the outmost importance, since our main goal is to ensure that AuNPs are cleared from the tumour sites only after laser activation and PTT session are concluded.

Biodistribution was assessed by quantifying the gold content (%, in relation to the injected dosage) in liver, kidney, and serum, after 24 h post-s.c. administration of HAOA-coated- and Holo-Tf-functionalized HAOA-coated AuNPs. Based on other studies, AuNPs’ biodistribution seems to be size-dependent. NPs with sizes greater than 200 nm tend to accumulate mainly in the liver, although they can also accumulate in the lungs, spleen, and kidneys [59,60]. Our results showed that the liver was indeed the organ with the greater gold accumulation (30%, for the HTf-functionalized HAOA-coated AuNPs), but virtually no gold has accumulated in the kidney and serum (>0.25%). Moreover, functionalizing the HAOA-coated AuNPs with HTF significantly reduced the gold content of the liver, from 60% (HAOA-coated AuNPs) to 30% (HTf-functionalized HAOA-coated AuNPs). The organs were also weighted, and tissue indexes of the main clearance and excretion organs were determined. Tissue indexes provide important information regarding chemically-induced changes to the analysed organs. Under physiological healthy circumstances, tissue indexes remained constant. However, increased tissue index suggests organ hypertrophy or congestion, whereas decreased tissue index may indicate that the organ may have undergone atrophy or any other degenerative changes [32,61]. HAOA-coated AuNPs caused a significant increase in the liver tissue index at 24 h post-administration. Moreover, HTf-functionalized HAOA-coated AuNPs administration also resulted in a significant increase of hepatic tissue index, both 4 h and 24 h after administration, although more pronounced at 24 h post-administration. The increase of index of the liver may be related with the higher expression of the TfR1/CD71 [62], which causes higher hepatic affinity for HTf-functionalized HAOA-coated AuNPs, thus leading to its accumulation in the liver. However, hepatic TfR1/CD71 expression does not fully explain the increased liver tissue index that resulted from HAOA-coated AuNPs administration. In fact, it has been described that when NPs’ surface is impregnated with Tf, its liver accumulation is not dependent on Tf content and that most nanoformulations accumulate in the liver, as part of their normal clearance and excretion processes [63,64]. This fact was also supported by the ICP-MS analysis results, that revealed that by functionalizing the AuNPs with HTf significantly reduced its hepatic accumulation. Furthermore, since no histological abnormalities in the liver were found, one can infer that the increased liver tissue index is related to their normal clearance process. Nevertheless, others have suggested that in tumours with high TfR1/CD71 expression, such as ATC, Tf-funcionalized NPs tend to accumulate more in the tumour itself than in the liver, when compared to the non-functionalized counterpart [63]. This aspect still need further testing with our HTf-functionalized HAOA-coated AuNPs, which we aim to do in the future, using in vivo murine models of ATC. Taking these results together, the HTf-functionalized HAOA-coated AuNPs are deemed as safe for in situ administration in vivo.

With all these results in mind, the HTf-functionalized HAOA-coated (5:10) AuNPs gathers all the required characteristics for the design of an ATC-specific AuNPs for NIR-PTT. Indeed, our functionalized coated NPs present a size between 200 nm and 1 µm. Concerning size and shape, the AuNPs also monodisperse, allowing the predictability of the formulations in vivo, pharmacological and physicochemical properties as well as how the AuNPs will interact with biological entities such as cells. Moreover, these NPs present spherical morphology, optimizing surface-area-to-volume ratio, and consequently cell internalization. Its SPR band is within the NIR wavelengths, and more specifically in the 800s’ nm, ensuring optimal activation by the laser emitting at 808 nm. The formulations are safe and non-haemolytic, as tested in vitro and in vivo; and finally, our ATC-specific AuNPs were selective and cytotoxic for ATC, causing a very significative decrease in cell viability of an ATC cell line, upon laser activation.

The study reported herein resulted in very exciting preliminary results of a formulation designed for NIR-PTT of ATC, and thus it should be continued. Taking solely these results into account, this formulation seems highly promising for a future possible treatment, as a palliative treatment to improve patients’ quality of life, as an adjuvant therapy to reduce the ATC mass and allow it to be surgically resettable, or yet as a monotherapy that would completely ablate ATC. Further studies will include the in vivo efficacy and safety assessments using ATC-bearing animal models. Undoubtedly, this data is crucial to developing better therapeutic approaches to this disease and to predict the course of treatment.

## 5. Conclusions

The current study explored the feasibility of a new formulation of functionalized AuNPs for PTT of ATC, one of the most lethal malignancies with no viable treatment course. Several combinations of ligands and AuNP cores were tested. Although all functionalized formulations were deemed safe for a healthy epithelial cell line and selective for ATC, the HTf-functionalized HAOA-coated (5:10) AuNPs was the formulation that presented the best set of characteristics suitable to specifically target ATC cells for PTT. Toxicity and biodistribution in vivo studies also rendered this formulation as safe, with no abnormalities of the analysed organs and reduced gold accumulation in organs.

Taking all the results reported into consideration, the innovative formulation developed by our group, the holo-Transferrin functionalized HAOA-coated AuNPs demonstrated to have all the desired physical characteristics for optimized in situ administration and NIR-PTT, and had very promising preliminary results regarding selectivity and efficacy in vitro, and in vivo and in vitro safety, for the PTT of ATC.

Although future work is still necessary, the preliminary results herein presented show that this is a very promising path to find a possible treatment of this hopeless disease.

## Figures and Tables

**Table 1:** Mean size, polydispersivity index (PdI), and recovery yield (RY) of the AuNPs, throughout the different steps of the formulation.

Samples		Functionalization (Ligands)	Mean Size (nm)	PdI
5:3 AuNPs	Uncoated		88.0 ± 11.6	0.319 ± 0.061
	HAOA-coated		125.3 ± 25.7	0.312 ± 0.072
5:10 AuNPs	Uncoated		314.7 ± 42.9 ***	0.475 ± 0.029
	HAOA-coated		354.0 ± 29.3	0.394 ± 0.176
	HAOA-coated	EGF	671.5 ± 65.8 **	0.579 ± 0.058
	HAOA-coated	HTf	415.3 ± 47.4	0.375 ± 0.078
	HAOA-coated	Lap	483.4 ± 179.7	0.494 ± 0.072

Data is presented as mean ±SD, *n* > 3 (** *p* < 0.01 vs. 5:10 AuNPs HAOA-coated, *** *p* < 0.001 vs. 5:3 AuNPs uncoated).

**Table 2:** SPR peak (Abs_max_) of both 5:3 and 5:10 AuNPs, throughout the different steps of the formulation.

Sample		SPR Band (Abs_max_ nm)
5:3 AuNPs	Uncoated	551 ± 26
	HAOA-coated	536 ± 2
5:10 AuNPs	Uncoated	753 ± 54
	HAOA-coated	840 ± 122
	EGF-HAOA-coated	785 ± 127
	HTf-HAOA-coated	710 ± 128
	Lap-HAOA-coated	481 ± 25

Data is presented as mean ± SD, *n* > 3.

**Table 3:** Conjugation efficiency (%) of functionalized HAOA-coated 5:10 gold:RA (m/m) ratio AuNPs.

Sample		Conjugation Efficiency (%)
HAOA-coated AuNPs	EGF-functionalized	77.5 ± 9.4
	HTf-functionalized	80.8 ± 8.7
	Lap-functionalized	81.0 ± 5.8

Mean value ± SD, *n* = 3.

**Table 4:** Tissue indexes (liver, spleen, kidneys, and lungs) of mice at 4 and 24 h post-s.c. administration of HAOA-coated and HTf-functionalized HAOA-coated AuNPs. Control represents the tissue indexes of untreated animals.

Treatment		Tissue Index			
		Liver	Spleen	Kidneys	Lungs
Control		20.1 ± 0.1	6.0 ± 0.4	9.7 ± 0.1	7.2 ± 0.2
HAOA-coated AuNPs	4 h	20.4 ± 0.4	5.8 ± 0.5	10.1 ± 0.2	7.9 ± 0.2
	24 h	21.6 ± 0.6 *	6.3 ± 0.6	10.4 ± 0.1	7.7 ± 0.2
HTf-functionalized HAOA-coated AuNPs	4 h	21.4 ± 0.1 *	6.7 ± 0.2	10.5 ± 0.1	7.6 ± 0.3
	24 h	21.7 ± 1.0 **	6.7 ± 0.2	10.5 ± 0.2	8.0 ± 0.3

Results are presented as mean ± SD, *n* = 3 (* *p* < 0.05, ** *p* < 0.01 vs. control).

## Data Availability

The data presented in this study are available on request from the corresponding author. The data are not publicly available due to privacy reasons.

## References

[1] Amaral M., Afonso R.A., Gaspar M.M., Reis C.P. (2020). Anaplastic thyroid cancer: How far can we go?. EXCLI J..

[2] Ragazzi M., Ciarrocchi A., Sancisi V., Gandolfi G., Bisagni A., Piana S. (2014). Update on Anaplastic Thyroid Carcinoma: Morphological, Molecular, and Genetic Features of the Most Aggressive Thyroid Cancer. Int. J. Endocrinol..

[3] Ferrari S.M., Elia G., Ragusa F., Ruffilli I., La Motta C., Paparo S.R., Patrizio A., Vita R., Benvenga S., Materazzi G. (2020). Novel treatments for anaplastic thyroid carcinoma. Gland Surg..

[4] Molinaro E., Romei C., Biagini A., Sabini E., Agate L., Mazzeo S., Materazzi G., Sellari-Franceschini S., Ribechini A., Torregrossa L. (2017). Anaplastic thyroid carcinoma: From clinicopathology to genetics and advanced therapies. Nat. Rev. Endocrinol..

[5] Saini S., Tulla K., Maker A.V., Burman K.D., Prabhakar B.S. (2018). Therapeutic advances in anaplastic thyroid cancer: A current perspective. Mol. Cancer.

[6] Hsu K., Yu X., Audhya A.W., Jaume J.C., Lloyd R.V., Miyamoto S., Prolla T.A., Chen H. (2014). Novel Approaches in Anaplastic Thyroid Cancer Therapy. Oncologist.

[7] Liu T.-R., Xiao Z.-W., Xu H.-N., Long Z., Wei F.-Q., Zhuang S.-M., Sun X.-M., Xie L.-E., Mu J.-S., Yang A.-K. (2016). Treatment and Prognosis of Anaplastic Thyroid Carcinoma: A Clinical Study of 50 Cases. PLoS ONE.

[8] Hartmann J., Haap M., Kopp H.-G., Lipp H.-P. (2009). Tyrosine Kinase Inhibitors—A Review on Pharmacology, Metabolism and Side Effects. Curr. Drug Metab..

[9] Huang X., El-Sayed M.A. (2011). Plasmonic photo-thermal therapy (PPTT). Alex. J. Med..

[10] Zou L., Wang H., He B., Zeng L., Tan T., Cao H., He X., Zhang Z., Guo S., Li Y. (2016). Current Approaches of Photothermal Therapy in Treating Cancer Metastasis with Nanotherapeutics. Theranostics.

[11] Xu L., Cheng L., Wang C., Peng R., Liu Z. (2014). Conjugated polymers for photothermal therapy of cancer. Polym. Chem..

[12] Shibu E.S., Hamada M., Murase N., Biju V. (2013). Nanomaterials formulations for photothermal and photodynamic therapy of cancer. J. Photochem. Photobiol. C Photochem. Rev..

[13] Huang X., Jain P.K., El-Sayed I.H., El-Sayed M.A. (2008). Plasmonic photothermal therapy (PPTT) using gold nanoparticles. Lasers Med. Sci..

[14] Jaque D., Martínez Maestro L., Del Rosal B., Haro-Gonzalez P., Benayas A., Plaza J.L., Martín Rodríguez E., García Solé J. (2014). Nanoparticles for photothermal therapies. Nanoscale.

[15] Beik J., Abed Z., Ghoreishi F.S., Hosseini-Nami S., Mehrzadi S., Shakeri-Zadeh A., Kamrava S.K. (2016). Nanotechnology in hyperthermia cancer therapy: From fundamental principles to advanced applications. J. Control. Release.

[16] Ashraf S., Pelaz B., Del Pino P., Carril M., Escudero A., Parak W.J., Soliman M.G., Zhang Q., Carrillo-Carrion C. (2016). Gold-Based Nanomaterials for Applications in Nanomedicine. Light-Responsive Nanostructured Systems for Applications in Nanomedicine. Topics in Current Chemistry.

[17] Zhang X. (2015). Gold Nanoparticles: Recent Advances in the Biomedical Applications. Cell Biochem. Biophys..

[18] Carabineiro S. (2017). Applications of Gold Nanoparticles in Nanomedicine: Recent Advances in Vaccines. Molecules.

[19] Riley R.S., Day E.S. (2017). Gold nanoparticle-mediated photothermal therapy: Applications and opportunities for multimodal cancer treatment. Wiley Interdiscip. Rev. Nanomed. Nanobiotechnol..

[20] Oyelere A. (2008). Gold nanoparticles: From nanomedicine to nanosensing. Nanotechnol. Sci. Appl..

[21] Hwang S., Nam J., Jung S., Song J., Doh H., Kim S. (2014). Gold nanoparticle-mediated photothermal therapy: Current status and future perspective. Nanomedicine.

[22] Mendes R., Pedrosa P., Lima J.C., Fernandes A.R., Baptista P.V. (2017). Photothermal enhancement of chemotherapy in breast cancer by visible irradiation of Gold Nanoparticles. Sci. Rep..

[23] Chung U.S., Kim J.-H., Kim B., Kim E., Jang W.-D., Koh W.-G. (2016). Dendrimer porphyrin-coated gold nanoshells for the synergistic combination of photodynamic and photothermal therapy. Chem. Commun..

[24] Wu P., Gao Y., Zhang H., Cai C. (2012). Aptamer-Guided Silver–Gold Bimetallic Nanostructures with Highly Active Surface-Enhanced Raman Scattering for Specific Detection and Near-Infrared Photothermal Therapy of Human Breast Cancer Cells. Anal. Chem..

[25] Zhou J., Lu Z., Zhu X., Wang X., Liao Y., Ma Z., Li F. (2013). NIR photothermal therapy using polyaniline nanoparticles. Biomaterials.

[26] Yang W., Liang H., Ma S., Wang D., Huang J. (2019). Gold nanoparticle based photothermal therapy: Development and application for effective cancer treatment. Sustain. Mater. Technol..

[27] Silva C.O., Rijo P., Molpeceres J., Ascensão L., Roberto A., Fernandes A.S., Gomes R., Pinto Coelho J.M., Gabriel A., Vieira P. (2016). Bioproduction of gold nanoparticles for photothermal therapy. Ther. Deliv..

[28] Silva C.O., Petersen S.B., Reis C.P., Rijo P., Molpeceres J., Fernandes A.S., Gonçalves O., Gomes A.C., Correia I., Vorum H. (2016). EGF Functionalized Polymer-Coated Gold Nanoparticles Promote EGF Photostability and EGFR Internalization for Photothermal Therapy. PLoS ONE.

[29] Lopes J., Coelho J.M.P., Vieira P.M.C., Viana A.S., Gaspar M.M., Reis C. (2020). Preliminary Assays towards Melanoma Cells Using Phototherapy with Gold-Based Nanomaterials. Nanomaterials.

[30] Saadat E. (2016). Development and Validation of Rapid RP-HPLC-DAD Analysis Method for Simultaneous Quantitation of Paclitaxel and Lapatinib in Polymeric Micelle Formulation. Sci. Pharm..

[31] Mota A.H., Andrade J.M., Rodrigues M.J., Custódio L., Bronze M.R., Duarte N., Baby A., Rocha J., Gaspar M.M., Simões S. (2020). Synchronous insight of in vitro and in vivo biological activities of Sambucus nigra L. extracts for industrial uses. Ind. Crops Prod..

[32] Pinho J.O., Amaral J.D., Castro R.E., Rodrigues C.M., Casini A., Soveral G., Gaspar M.M. (2019). Copper complex nanoformulations featuring highly promising therapeutic potential in murine melanoma models. Nanomedicine.

[33] ISO 10993-5:2009(E) Biological Devices—Part 5: Tests for In Vitro Cytotoxicity. http://nhiso.com/wp-content/uploads/2018/05/ISO-10993-5-2009.pdf.

[34] Herizchi R., Abbasi E., Milani M., Akbarzadeh A. (2016). Current methods for synthesis of gold nanoparticles. Artif. Cells Nanomed. Biotechnol..

[35] Allen J.M., Xu J., Blahove M., Canonico-May S.A., Santaloci T.J., Braselton M.E., Stone J.W. (2017). Synthesis of less toxic gold nanorods by using dodecylethyldimethylammonium bromide as an alternative growth-directing surfactant. J. Colloid Interface Sci..

[36] Chaloupka K., Malam Y., Seifalian A.M. (2010). Nanosilver as a new generation of nanoproduct in biomedical applications. Trends Biotechnol..

[37] Biterge-Süt B., Canpolat E. (2019). Evaluation of Gold Nanoparticles in Terms of Their Use in Biomedical Applications. Turk. J. Agric. Food Sci. Technol..

[38] El Halabi I., Bejjany R., Nasr R., Mukherji D., Temraz S., Nassar F., El Darsa H., Shamseddine A. (2018). Ascorbic Acid in Colon Cancer: From the Basic to the Clinical Applications. Int. J. Mol. Sci..

[39] Ngo Y.L., Lau C.H., Chua L.S. (2018). Review on rosmarinic acid extraction, fractionation and its anti-diabetic potential. Food Chem. Toxicol..

[40] Da Silva S.B., Amorim M., Fonte P., Madureira R., Ferreira D., Pintado M., Sarmento B. (2015). Natural extracts into chitosan nanocarriers for rosmarinic acid drug delivery. Pharm. Biol..

[41] Bragta P., Sidhu R.K., Jyoti K., Baldi A., Jain U.K., Chandra R., Madan J. (2018). Intratumoral administration of carboplatin bearing poly (ε-caprolactone) nanoparticles amalgamated with in situ gel tendered augmented drug delivery, cytotoxicity, and apoptosis in melanoma tumor. Colloids Surf. B Biointerfaces.

[42] Holback H., Yeo Y. (2011). Intratumoral Drug Delivery with Nanoparticulate Carriers. Pharm. Res..

[43] Öztürk A.A., Yenilmez E., Özarda M.G. (2019). Clarithromycin-Loaded Poly (Lactic-co-glycolic Acid) (PLGA) Nanoparticles for Oral Administration: Effect of Polymer Molecular Weight and Surface Modification with Chitosan on Formulation, Nanoparticle Characterization and Antibacterial Effects. Polymers.

[44] Verma A., Stellacci F. (2010). Effect of Surface Properties on Nanoparticle-Cell Interactions. Small.

[45] Robertson J.D., Rizzello L., Avila-Olias M., Gaitzsch J., Contini C., Magoń M.S., Renshaw S.A., Battaglia G. (2016). Purification of Nanoparticles by Size and Shape. Sci. Rep..

[46] Hwang N.-M., Jung J.-S., Lee D.-K. (2012). Thermodynamics and Kinetics in the Synthesis of Monodisperse Nanoparticles. Thermodynamics—Fundamentals and Its Application in Science.

[47] Vines J.B., Yoon J.-H., Ryu N.-E., Lim D.-J., Park H. (2019). Gold Nanoparticles for Photothermal Cancer Therapy. Front. Chem..

[48] Maciulevičius M., Vinčiūnas A., Brikas M., Butsen A., Tarasenka N., Tarasenko N., Račiukaitis G. (2013). Pulsed-laser generation of gold nanoparticles with on-line surface plasmon resonance detection. Appl. Phys. A.

[49] Schiff B.A. (2004). Epidermal Growth Factor Receptor (EGFR) Is Overexpressed in Anaplastic Thyroid Cancer, and the EGFR Inhibitor Gefitinib Inhibits the Growth of Anaplastic Thyroid Cancer. Clin. Cancer Res..

[50] Ensinger C., Spizzo G., Moser P., Tschoerner I., Prommegger R., Gabriel M., Mikuz G., Schmid K.W. (2004). Epidermal Growth Factor Receptor as a Novel Therapeutic Target in Anaplastic Thyroid Carcinomas. Ann. N. Y. Acad. Sci..

[51] Elliott D.D., Sherman S.I., Busaidy N.L., Williams M.D., Santarpia L., Clayman G.L., El-Naggar A.K. (2008). Growth factor receptors expression in anaplastic thyroid carcinoma: Potential markers for therapeutic stratification. Hum. Pathol..

[52] Lee D.H., Lee G.K., Kong S.-Y., Kook M.C., Yang S.K., Park S.Y., Park S.H., Keam B., Park D.J., Cho B.Y. (2006). Epidermal growth factor receptor status in anaplastic thyroid carcinoma. J. Clin. Pathol..

[53] Magro G., Cataldo I., Amico P., Torrisi A., Vecchio G.M., Parenti R., Asioli S., Recupero D., D’Agata V., Mucignat M.T. (2011). Aberrant Expression of TfR1/CD71 in Thyroid Carcinomas Identifies a Novel Potential Diagnostic Marker and Therapeutic Target. Thyroid.

[54] Parenti R., Salvatorelli L., Magro G. (2014). Anaplastic Thyroid Carcinoma: Current Treatments and Potential New Therapeutic Options with Emphasis on TfR1/CD71. Int. J. Endocrinol..

[55] Campisi A., Bonfanti R., Raciti G., Bonaventura G., Legnani L., Magro G., Pennisi M., Russo G., Chiacchio M.A., Pappalardo F. (2020). Gene Silencing of Transferrin-1 Receptor as a Potential Therapeutic Target for Human Follicular and Anaplastic Thyroid Cancer. Mol. Ther. Oncolytics.

[56] Liebner D.A., Haraldsdottir S., Shah M.H. (2016). Potential Approaches to Chemotherapy of Thyroid Cancer in the Future. Thyroid Cancer.

[57] Amin K., Dannenfelser R.-M. (2006). In vitro hemolysis: Guidance for the pharmaceutical scientist. J. Pharm. Sci..

[58] Gustafson H.H., Holt-Casper D., Grainger D.W., Ghandehari H. (2015). Nanoparticle uptake: The phagocyte problem. Nano Today.

[59] De Jong W.H., Hagens W.I., Krystek P., Burger M.C., Sips A.J.A.M., Geertsma R.E. (2008). Particle size-dependent organ distribution of gold nanoparticles after intravenous administration. Biomaterials.

[60] Sonavane G., Tomoda K., Makino K. (2008). Biodistribution of colloidal gold nanoparticles after intravenous administration: Effect of particle size. Colloids Surf. B Biointerfaces.

[61] Nave M., Castro R.E., Rodrigues C.M., Casini A., Soveral G., Gaspar M.M. (2016). Nanoformulations of a potent copper-based aquaporin inhibitor with cytotoxic effect against cancer cells. Nanomedicine.

[62] Anderson G.J., Frazer D.M. (2005). Hepatic Iron Metabolism. Semin. Liver Dis..

[63] Choi C.H.J., Alabi C.A., Webster P., Davis M.E. (2010). Mechanism of active targeting in solid tumors with transferrin-containing gold nanoparticles. Proc. Natl. Acad. Sci. USA.

[64] Bartlett D.W., Su H., Hildebrandt I.J., Weber W.A., Davis M.E. (2007). Impact of tumor-specific targeting on the biodistribution and efficacy of siRNA nanoparticles measured by multimodality in vivo imaging. Proc. Natl. Acad. Sci. USA.

